# Diagnosing Sarcopenia with AI-Aided Ultrasound (DINOSAUR)—A Pilot Study

**DOI:** 10.3390/nu16162768

**Published:** 2024-08-20

**Authors:** Vanessa Yik, Shawn Shi Xian Kok, Esther Chean, Yi-En Lam, Wei-Tian Chua, Winson Jianhong Tan, Fung Joon Foo, Jia Lin Ng, Sharmini Sivarajah Su, Cheryl Xi-Zi Chong, Darius Kang-Lie Aw, Nathanelle Ann Xiaolian Khoo, Paul E. Wischmeyer, Jeroen Molinger, Steven Wong, Lester Wei-Lin Ong, Frederick Hong-Xiang Koh

**Affiliations:** 1 Duke-NUS Medical School, Singapore 169857, Singapore; vanessa.yik@u.duke.nus.edu; 2 Department of Radiology, Sengkang General Hospital, Singapore 544886, Singapore; 3 Department of Colorectal Surgery, Sengkang General Hospital, Singapore 544886, Singapore; 4 Department of Anesthesia, Duke University Medical Center, Durham, NC 27710, USA; 5 Department of Anesthesia, Human Pharmacology and Physiology Lab (HPPL), Duke University Medical Center, Durham, NC 27710, USA; 6 Department of Intensive Care Adults, Erasmus Medical Center University, 3015 GD Rotterdam, The Netherlands; 7 Department of Surgery, Sengkang General Hospital, Singapore 544886, Singapore; 8 Lee Kong Chian School of Medicine, Nanyang Technological University, Singapore 308232, Singapore

**Keywords:** sarcopenia, muscle quality, intramuscular adipose tissue, artificial intelligence, ultrasound

## Abstract

**Background:** Sarcopenia has been recognized as a determining factor in surgical outcomes and is associated with an increased risk of postoperative complications and readmission. Diagnosis is currently based on clinical guidelines, which includes assessment of skeletal muscle mass but not quality. Ultrasound has been proposed as a useful point-of-care diagnostic tool to assess muscle quality, but no validated cut-offs for sarcopenia have been reported. Using novel automated artificial intelligence (AI) software to interpret ultrasound images may assist in mitigating the operator-dependent nature of the modality. Our study aims to evaluate the fidelity of AI-aided ultrasound as a reliable and reproducible modality to assess muscle quality and diagnose sarcopenia in surgical patients. **Methods:** Thirty-six adult participants from an outpatient clinic were recruited for this prospective cohort study. Sarcopenia was diagnosed according to Asian Working Group for Sarcopenia (AWGS) 2019 guidelines. Ultrasonography of the rectus femoris muscle was performed, and images were analyzed by an AI software (MuscleSound® (Version 5.69.0)) to derive muscle parameters including intramuscular adipose tissue (IMAT) as a proxy of muscle quality. A receiver operative characteristic (ROC) curve was used to assess the predictive capability of IMAT and its derivatives, with area under the curve (AUC) as a measure of overall diagnostic accuracy. To evaluate consistency between ultrasound users of different experience, intra- and inter-rater reliability of muscle ultrasound parameters was analyzed in a separate cohort using intraclass correlation coefficients (ICC) and Bland–Altman plots. **Results:** The median age was 69.5 years (range: 26–87), and the prevalence of sarcopenia in the cohort was 30.6%. The ROC curve plotted with IMAT index (IMAT% divided by muscle area) yielded an AUC of 0.727 (95% CI: 0.551–0.904). An optimal cut-off point of 4.827%/cm^2^ for IMAT index was determined with a Youden’s Index of 0.498. We also demonstrated that IMAT index has excellent intra-rater reliability (ICC = 0.938, CI: 0.905–0.961) and good inter-rater reliability (ICC = 0.776, CI: 0.627–0.866). In Bland–Altman plots, the limits of agreement were from −1.489 to 1.566 and −2.107 to 4.562, respectively. **Discussion:** IMAT index obtained via ultrasound has the potential to act as a point-of-care evaluation for sarcopenia screening and diagnosis, with good intra- and inter-rater reliability. The proposed IMAT index cut-off maximizes sensitivity for case finding, supporting its use as an easily implementable point-of-care test in the community for sarcopenia screening. Further research incorporating other ultrasound parameters of muscle quality may provide the basis for a more robust diagnostic tool to help predict surgical risk and outcomes.

## 1. Introduction

Sarcopenia is the age-related decline in skeletal muscle mass and loss of muscle strength, with or without reduced physical performance [1]. With an estimated global prevalence of up to 27% [2], it is a significant component of clinical frailty and is regarded as a predictor of morbidity, disability, and death in the elderly [3].

As the world’s population continues to age, the prevalence of sarcopenia is expected to increase—in Singapore, a soon to be “super-aged” country, one in four citizens will be aged 65 and above by 2030 [4]. With the advent of better surgical techniques and increased life expectancy, there has been a steady growth of surgical procedures performed in geriatric patients [5,6]. Sarcopenia has been recognized as a determining factor in the success of the surgery and is associated with an increased risk of postoperative complications and hospital readmission [7]. For this reason, there is great interest in the ability to detect and optimize patients with sarcopenia prior to surgery to reduce its impact on patients’ health and outcomes [7] as well as curb the growing socioeconomic burden of healthcare costs.

Myosteatosis—skeletal muscle fat infiltration—is a significant contributing factor to sarcopenia and a hallmark of poor muscle quality. Muscle quality, not just quantity, has been recognized as an important marker of sarcopenia. Intramuscular adipose tissue (IMAT), a measure of muscle quality, has been closely correlated to reduced muscle strength [8] and identified as a significant risk factor for postoperative complications [9] and cardiorespiratory fitness [10,11,12].

Currently, sarcopenia is diagnosed based on clinical guidelines such as those established by the Asian Working Group for Sarcopenia (AWGS) in 2019, with components comprising muscle strength, physical performance, and skeletal muscle mass that aim to assess muscle quality and quantity [1]. AWGS 2019 specifies sarcopenic cut-offs for appendicular skeletal muscle mass measured by dual-energy X-ray absorptiometry (DEXA) and bioelectrical impedance analysis (BIA). DEXA uses low amounts of radiation and concurrently provides measurements of lean mass, fat mass, and bone mineral content, while BIA measures the impedance of a low-intensity electrical current through the body to calculate muscle mass [13]. However, their results can be greatly affected by the hydration or exercise status of the subject, and these modalities are also majorly limited in their assessment of muscle quality, such as the degree of fat infiltration in muscle [13,14].

Due to the limitations of DEXA and BIA, there has been increasing interest in the use of other imaging modalities—computed tomography (CT), magnetic resonance imaging (MRI), and ultrasound—to assess muscle quality. Features of muscle quality like IMAT are conventionally assessed via cross-sectional imaging such as CT and MRI, but they require radiological expertise to interpret, are costly, and are not routine in clinical practice. Conversely, ultrasonography is an inexpensive, widely available, radiation-free alternative that has been gaining traction in the assessment of muscle quality [15], particularly in athletes [15], children [16], and critically ill adults [17]. It is strongly correlated to CT- and MRI-based muscle measurements [18]. Yet, current sarcopenia guidelines do not include cut-offs for ultrasound assessment for diagnosis. The main limitation of the use of ultrasound is its lack of standardization and the partly operator-dependent quality of implementation, with high variability between assessors [14]. However, the development of novel automated annotation technology to interpret ultrasound images with build-in guidance [17], such as the MuscleSound^®^ (Version 5.69.0) (Denver, CO, USA) software, has the potential to mitigate these shortcomings while providing a standardized technique to evaluate muscle quality that can be easily picked up by novice users and implemented in practice.

Thus far, the clinical utility of AI-aided muscle ultrasound in surgical patients in an Asian cohort has not been reported. Hence, there remains a need for a validated point-of-care tool to assess muscle quality for sarcopenia diagnosis that is reliable, inexpensive, and easy to use. This study aims to address these needs by first identifying ultrasound-specific sarcopenia cut-offs, and second, assessing the inter- and intra-rater reliability of AI-aided ultrasound results between novice and experienced users.

## 2. Materials and Methods

### 2.1. Study Design and Participants

A single-center prospective cohort study was conducted with approval by the SingHealth Centralized Institutional Review Board (CIRB #: 2022/2027) between August and December 2023. Adult patients aged 21–90 who were seen at the surgical clinic and planned for elective major gastrointestinal surgery at Sengkang General Hospital were recruited for this study. This patient population receives standardized peri-operative sarcopenia assessment at our institution and was selected for the study due to the higher prevalence of sarcopenia compared to the community-dwelling community [19]. Exclusion criteria included patients with cancer cachexia (defined as weight loss ≥10% from usual body weight and presence of at least one symptom of anorexia, fatigue, or early satiation [20]) or disease-related factors (such as tumor crisis or advanced cancer). Patients who were non-ambulant or whose rectus femoris were unable to be assessed were also excluded.

Eligible patients were enrolled from the outpatient clinic at Sengkang General Hospital, and written informed consent was sought. As part of routine pre-operative assessments, the participants were evaluated for sarcopenia by trained research coordinators according to the AWGS 2019 diagnostic criteria, comprising three components: handgrip strength (muscle strength), gait speed (physical performance), and BIA (appendicular skeletal muscle mass) (Table 1). Handgrip strength was measured using a hand dynamometer on the dominant hand. Gait speed was measured by conducting a 6-m walk test. Height and weight were also measured to determine body mass index (BMI).

Using the expected prevalence of sarcopenia of about 40% in the hospital population [21] and with an expected minimum test sensitivity of 90% and specificity of 80% based on previous studies on other ultrasound parameters [22,23], with a type I error rate of 5%, power of 80%, and a drop-out rate of 10%, a sample size of 36 was required.

### 2.2. Muscle Ultrasonography Procedure

**Figure 1:**
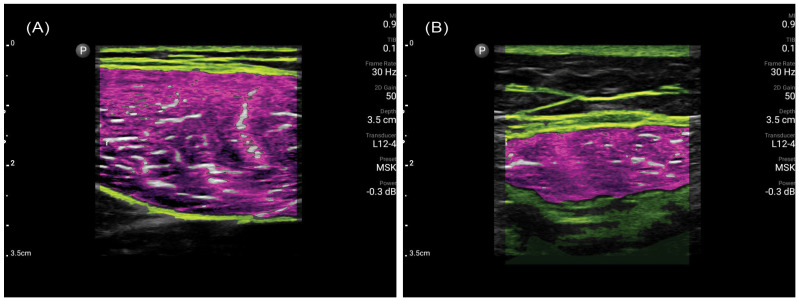
Typical ultrasound images obtained of the rectus femoris muscle after analysis by MuscleSound^®^. (**A**) is a scan from a 27-year-old volunteer, and (**B**) is from an 85year-old patient. The pink overlay represents the muscle, yellow represents the muscle boundary and white highlights are non-contractile fibers (in color for print).

In the same sitting, a trained ultrasound user (either a musculoskeletal sonographer or radiologist) performed ultrasonography of the bilateral rectus femoris (RF) muscles with the patient lying on a flat examination bed. The probe placement at the midpoint of the RF was standardized by the MuscleSound^®^ software based on the patient’s height. Ultrasound images were obtained using a portable ultrasound system (Philips Lumify, Amsterdam, The Netherlands) with a linear array transducer (4–12 MHz, 34 mm aperture size, Lumify L12-4 Android, Amsterdam, The Netherlands) on the musculoskeletal exam preset. The images were then uploaded to MuscleSound^®^ for analysis. MuscleSound^®^ is an automated annotation software with a proprietary artificial intelligence (AI) algorithm to analyze muscle ultrasound images, allowing rapid derivation of RF muscle parameters, including muscle thickness, IMAT, and IMAT index (IMAT divided by muscle area). Typical ultrasound images analyzed by MuscleSound^®^ are demonstrated in Figure 1. Ultrasound RF IMAT values were automatically calculated by MuscleSound^®^ using the ultrasound echo intensity in the equation published by Young et al. [24]. The average of left and right RF ultrasound results was used for all the analyses.

### 2.3. Intra- and Inter-Rater Reliability

Intra- and inter-rater reliability of muscle ultrasound parameters was analyzed in a separate cohort of adult participants recruited from the outpatient clinic and healthy volunteers to ensure a range of muscle quality within the study sample. Ultrasonography of the RF was performed bilaterally using the same Lumify portable ultrasound system and MuscleSound^®^ software by two blinded sonographers (a novice and an experienced sonographer) consecutively. The novice sonographer was a medical trainee with no prior formal ultrasound training, while the experienced sonographer was a practicing consultant radiologist with experience in musculoskeletal ultrasound. Both underwent orientation and training for the MuscleSound^®^ software to standardize the ultrasound technique. To assess the inter-rater reliability of MuscleSound^®^, the ultrasound results obtained by the novice and experienced sonographer were compared. Intra-rater reliability was assessed by comparing the readings of three consecutive sessions from each subject obtained by the novice sonographer.

Using the intraclass correlation coefficient hypothesis testing method [25], with a minimum expected reliability of 80%, expected reliability of 90%, alpha = 0.05, and power of 80%, a minimum sample size of 61 subjects was calculated to sufficiently determine the intra- and inter-rater reliability of MuscleSound^®^ in evaluating IMAT and its derivatives.

### 2.4. Statistical Analyses

Statistical analyses were performed using GraphPad Prism (Version 10.1.1) and IBM SPSS Statistics (Version 29.0.1). Study participant demographic characteristics and anthropometric measurements were summarized, using median (range) for continuous variables and percentages for categorical variables.

Receiver operative characteristic (ROC) analysis was used to assess the predictive capability of IMAT and its derivatives, with the area under the ROC curve (AUC) used as a measure of overall diagnostic accuracy. The sensitivity, specificity, and positive predictive and negative predictive values were calculated for a clinically relevant range of cut-off values. An AUC value of 0.7–0.8 was considered acceptable, 0.8–0.9 as excellent, and more than 0.9 as outstanding [26]. A *p*-value of <0.05 was considered statistically significant. Youden’s index was used to identify statistically optimal cut-offs for sarcopenia screening and diagnosis by comparing the sensitivity and specificity over a range of clinically relevant IMAT cut-offs.

Intra- and inter-rater reliability of muscle ultrasound parameters was analyzed using intraclass correlation coefficients (ICC) with a two-way mixed-effects model. Both the absolute agreement and consistency definitions were used to analyze inter-rater reliability to account for potential systematic errors. An ICC value between 0.75–1 was interpreted as excellent inter-rater agreement, between 0.6–0.74 as good, between 0.4–0.59 as fair, and <0.4 as poor, according to guidelines by Cicchetti [27]. Bland–Altman limits of agreement were plotted to visualize agreement between intra- and inter-rater measurements and identify possible proportional bias.

## 3. Results

### 3.1. Patient Characteristics

A total of 36 patients were recruited and included in the analysis. The median age was 69.5 years (range: 26–87), the median BMI was 23.11 kg/m^2^ (range: 16.94–33.21), and 17 (47.2%) participants were male. Based on the AWGS 2019 diagnostic criteria, the prevalence of sarcopenia in the cohort was 30.6% (Table 2).

### 3.2. Diagnostic Capability

**Figure 2:**
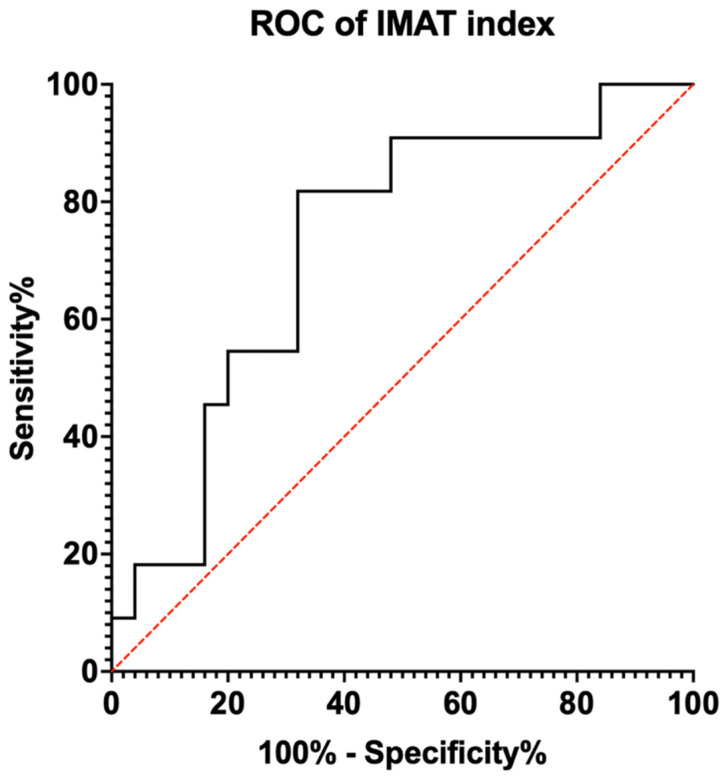
Receiver operating characteristic (ROC) curve for IMAT index with area under the curve. IMAT: intramuscular adipose tissue (in color for print).

ROC analysis was performed to evaluate the relationship between muscle ultrasound parameters and sarcopenia diagnosis. The AUC value for IMAT was AUC 0.553 (95% CI: 0.345–0.760), with a *p*-value not statistically significant at 0.619. For the IMAT index, the AUC value was 0.727 (95% CI: 0.551–0.904) with a *p*-value of 0.032 (Figure 2). Using the Youden’s Index, the optimal cut-off value for the IMAT index was determined to be 4.8265 %/cm^2^ (Table 3). Subgroup analysis by patient gender did not yield a higher AUC for the IMAT index.

### 3.3. Intra- and Inter-Rater Reliability

Sixty-one participants were recruited in the separate cohort to assess the intra- and inter-rater reliability of MuscleSound^®^ results. The median age was 27 years (range: 20–81), the median BMI was 22.7kg/m^2^ (range: 15.82–39.21), and 24 (39.3%) participants were male.

**Figure 3:**
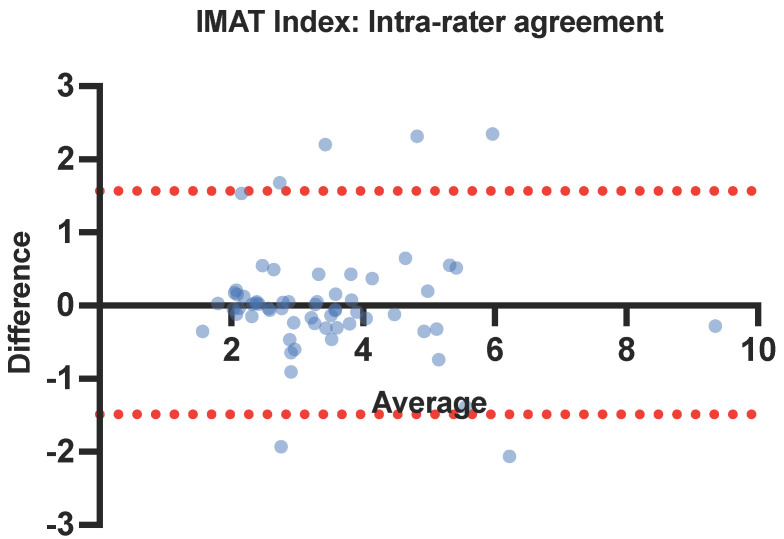
Bland–Altman plot for IMAT index intra-rater agreement. IMAT: intramuscular adipose tissue (in color for print).

The intra-rater ICCs of IMAT and the IMAT index were 0.824 (95% CI: 0.731–0.888) and 0.938 (95% CI: 0.905–0.961), respectively (Table 4). In Bland–Altman plots for the IMAT index intra-rater agreement, the average bias was 0.039, and the 95% limits of agreement were from −1.489 to 1.566 (Figure 3).

**Figure 4:**
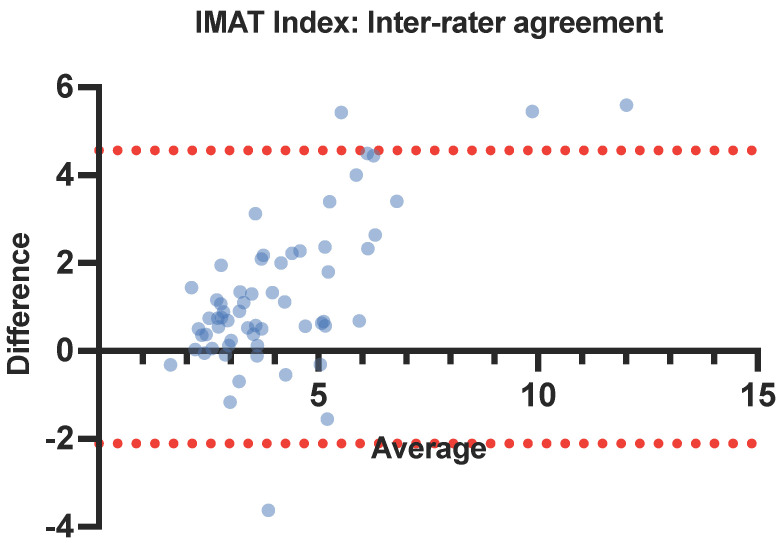
Bland–Altman plot for IMAT index inter-rater agreement. IMAT: intramuscular adipose tissue (in color for print).

The inter-rater ICCs of IMAT and the IMAT index were 0.623 (95% CI: 0.377–0.771) and 0.698 (95% CI: 0.284–0.852), respectively, using an absolute agreement definition. With a consistency definition, the inter-rater ICCs of IMAT and the IMAT index were 0.631 (95% CI: 0.385–0.779) and 0.776 (95% CI: 0.627–0.866), respectively (Table 5). In Bland–Altman plots for the IMAT index inter-rater agreement, the average bias was 1.23 and 95% limits of agreement were from −2.107 to 4.562 (Figure 4).

## 4. Discussion

The present study is the first to investigate the use of AI-aided ultrasound in assessing sarcopenia in an Asian cohort. The results demonstrate that the IMAT index derived from ultrasound has the potential to be a useful bedside tool for sarcopenia screening and diagnosis, with excellent intra-rater and good inter-rater reliability.

### 4.1. Efficient—Intra-Rater Variability

AI-aided ultrasound is a precise modality to assess muscle quality, with our results demonstrating excellent intra-rater reliability for both IMAT and IMAT index (ICC 0.824 and 0.938, respectively) derived from MuscleSound^®^. While some studies have previously evaluated the intra-rater consistency for other MuscleSound^®^-derived parameters [28,29] and manual intramuscular fat calculations [30], this study is the first to do so for AI-calculated IMAT. In the pursuit of determining ultrasound cut-offs for sarcopenia, standards for sonographic assessment protocols must be established. From our results, we propose that a single ultrasound scan of each RF with the AI program is sufficient to obtain representative IMAT results, enabling an efficient method to assess muscle quality.

### 4.2. Reliable—Inter-Rater Variability

There is good inter-rater reliability between an experienced and novice sonographer using AI-aided ultrasound to assess the IMAT index (ICC 0.776). IMAT index is normalized for muscle cross-sectional area as a correction, potentially taking into account small user variations in probe placement or scanning technique. In both intra- and inter-rater reliability, the IMAT index had a superior ICC compared to IMAT. Our results also suggest a positive bias between novice and experienced sonographers, as demonstrated in the IMAT index Bland–Altman plot. The IMAT index obtained by the experienced sonographer tended to be higher, possibly due to differences in scanning technique that affect the detected muscle area, such as pressure applied by the ultrasound probe.

Ultrasound is traditionally regarded as an operator-dependent modality, with associated limitations in image acquisition, measurements, and interpretation [8]. An automated program to derive IMAT values using validated algorithms is able to mitigate the potential user differences from the manual calculation and interpretation of ultrasound images. However, the AI guidance provided by MuscleSound^®^ in our study was not able to overcome all the variations in image acquisition between a novice and experienced user. Future studies may explore the learning curve for novice MuscleSound^®^ users and how it compares to traditional ultrasonography applications. A shallow learning curve for an operator-independent imaging system is ideal for rapid uptake and widespread use in the peri-operative setting as well as implementation at the community healthcare level to assess patients’ muscle quality and diagnose sarcopenia easily.

### 4.3. Accurate—Ultrasound for Sarcopenia Diagnosis

Our study also aimed to assess the predictive capability of IMAT and its derivatives, which were assessed by ultrasound with AI guidance for sarcopenia diagnosis. IMAT, a marker of myosteatosis [8], was chosen as a clinically significant parameter as it is correlated with muscle strength, functionality, and mortality in hospitalized geriatric patients [31] and is recognized as a risk factor for postoperative complications [9,32,33]. With an acceptable AUC of 0.727, the IMAT index has the potential to be used as a screening and diagnostic tool for sarcopenia [26]. Several potential cut-offs were identified, and an IMAT index of 4.8265 %/cm^2^ was selected to optimize sarcopenia case finding as a point-of-care screening test, corresponding to the maximum Youden’s Index of 0.498. Increased sarcopenia case finding will aid in earlier diagnosis, allowing for earlier intervention through physical activity, prehabilitation, and nutritional supplementation [34]. By recognizing and tackling sarcopenia in the preoperative window, patients can increase their physiological reserves through prehabilitation before undergoing the anesthetic and surgical stress of a major operative intervention [35].

Ultrasound has been gaining traction as a low-cost alternative for sarcopenia diagnosis [36,37], with potential applications in the community and primary care setting. The existing literature on ultrasound assessment of muscle has largely focused on parameters related to muscle quantity, such as muscle thickness, fascicle length, and pennation angle [37,38,39]. However, muscle quality, not just quantity, is becoming increasingly recognized as an important independent variable affecting patient outcomes [40]. Among the imaging modalities available, CT and MRI are unsuitable for repeated patient monitoring for progress, as they are time-consuming and have prohibitive cost barriers, and CT scans expose patients to unnecessary radiation [37]. In comparison, AI-guided portable ultrasound is an inexpensive, fast, and reliable method to assess muscle quality and screen for sarcopenia. Additional refinement to our muscle assessment protocol may yield an improvement in AUC, supporting the clinical utility of AI-guided ultrasound.

## 5. Limitations

While our results show promise in supporting the use of AI-aided ultrasound to assess sarcopenia, several limitations must be considered. The study utilizes the current gold-standard AWGS 2019 guidelines for the diagnosis of sarcopenia. However, it does not compare the ultrasound-derived muscle parameters with equivalent variables measured from CT or MRI. The use of ethnicity-specific guidelines may also affect the generalizability of our results.

As a pilot study, additional internal and external validation is needed to verify the results of our study. The wide age range and use of a surgical cohort, which may be a potential source of bias, would benefit from additional validation in populations with other disease conditions as well as in community-dwelling individuals. Additionally, the study’s cross-sectional nature limits our ability to evaluate the fidelity of AI-aided ultrasound in monitoring sarcopenia progression or establish causality between IMAT results and modifiable sarcopenia risk factors. Future studies should explore the changes in muscle quality over time and under various stresses by using muscle ultrasound to track patients through their surgical journey and the peri-operative period. Correlation of ultrasound muscle parameters with quality-of-life measures and functional outcomes will provide further insight into the impact of poor muscle quality and inform future strategies to combat sarcopenia. Other muscles or a composite of various representative muscle sites, such as gastrocnemius and rectus muscles, may be investigated as other potential sites for IMAT derivation.

## 6. Conclusions

IMAT index derived from AI-aided ultrasound has the potential to be a useful bedside tool for sarcopenia screening and diagnosis that is precise and operator-independent. The proposed IMAT index cut-off maximizes sensitivity for case finding, making it useful as a point-of-care test in the community for sarcopenia screening and enabling earlier intervention. Good intra- and inter-rater reliability between novice and experienced users further supports a push for implementation in the community. Additional studies are required to assess the feasibility of AI-aided ultrasound to track patients’ progress and muscle quality through various stages of treatment, such as the pre-, peri-, and post-operative period for surgical patients.

## Figures and Tables

**Table 1:** Asian Working Group for Sarcopenia (AWGS) 2019 diagnostic guidelines. M: male; F: female; m/s: meters per second; s: seconds; ASM: appendicular skeletal mass; kg/m^2^: kilograms per meter squared.

**Muscle strength**	
Handgrip strength	M: <28 kg, F: <18 kg
**Physical performance**	
6-meter walk	<1.0 m/s
*or* 5-time chair stand test	≥12 s
*or* Short Physical Performance Battery	≤9
**Appendicular skeletal muscle mass (ASM)**	
Dual-energy X-ray-absorptiometry	M: <7.0 kg/m^2^, F: <5.4 kg/m^2^
Bioelectrical impedance analysis	M: <7.0 kg/m^2^, F: <5.7 kg/m^2^
**Sarcopenia**	Low ASM + Low Muscle Strength **OR** Low Physical Performance
**Severe sarcopenia**	Low ASM + Low Muscle Strength **AND** Low Physical Performance

**Table 2:** Study patient characteristics. BMI: body mass index.

Patient Characteristics	Total *n* = 36
Age in years, median (range)	69.5 (26–81)
Male sex, *n* (%)	17 (47.2%)
BMI (kg/m^2^), median (range)	23.1 (16.8–33.2)
Height (m), median (range)	1.61 (1.31–1.74)
Weight (kg), median (range)	55 (39–88)
Sarcopenia, *n* (%)	11 (30.6%)

**Table 3:** Cut-off values and Youden’s index.

Cut-Off Value	Sensitivity	1—Specificity	Youden’s Index
2.7938	1.000	0.840	0.160
2.9705	0.909	0.760	0.149
3.3220	0.909	0.640	0.269
3.6830	0.909	0.560	0.349
3.7703	0.909	0.520	0.389
3.9598	0.909	0.480	0.429
4.6990	0.818	0.400	0.418
4.7275	0.818	0.360	0.458
**4.8265**	**0.818**	**0.320**	**0.498**
5.0920	0.727	0.320	0.407
5.3387	0.636	0.320	0.316
5.5428	0.545	0.280	0.265
5.7083	0.545	0.200	0.345
6.0910	0.455	0.200	0.255
6.9270	0.364	0.160	0.113
7.3528	0.182	0.160	0.022
7.8985	0.182	0.120	0.062

**Table 4:** Intra-rater IMAT and IMAT index. IMAT: intramuscular adipose tissue; RF: rectus femoris; ICC: intraclass correlation coefficients; CI: confidence interval. *: *p*-value < 0.05.

	Muscle Parameter			Intra-Rater Reliability		
	Session 1	Session 2	Session 3	ICC	95% CI	*p*-Value
**RF IMAT (%)**	15.1 (3.12)	14.6 (3.36)	14.6 (3.26)	0.824	0.781–0.888	<0.001 *
**RF IMAT index (%/cm^2^)**	3.48 (1.4)	3.44 (1.42)	3.36 (1.31)	0.938	0.905–0.961	<0.001 *

**Table 5:** Inter-rater IMAT and IMAT index. IMAT: intramuscular adipose tissue; RF: rectus femoris; ICC: intraclass correlation coefficients; CI: confidence interval. *: *p*-value < 0.05.

	Muscle Parameter		Inter-Rater Reliability		
	User 1	User 2	ICC	95% CI	*p*-Value
**RF IMAT (%)**	14.3 (3.22)	15.1 (3.12)	0.631	0.377–0.77	<0.001 *
**RF IMAT index (%/cm^2^)**	4.7 (2.44)	3.48 (1.4)	0.776	0.284–0.852	<0.001 *

## Data Availability

Due to institutional policy, the raw data supporting the conclusions of this article will be made available by the authors on request.
